# Supplementary figures and images for: Loss of Septation Initiation Network (SIN) kinases blocks tissue invasion and unlocks echinocandin cidal activity against Aspergillus fumigatus
Source: PLoS Pathog. 2021 Aug 9;17(8):e1009806. doi: 10.1371/journal.ppat.1009806 (PMC8376064; doi:10.1371/journal.ppat.1009806)

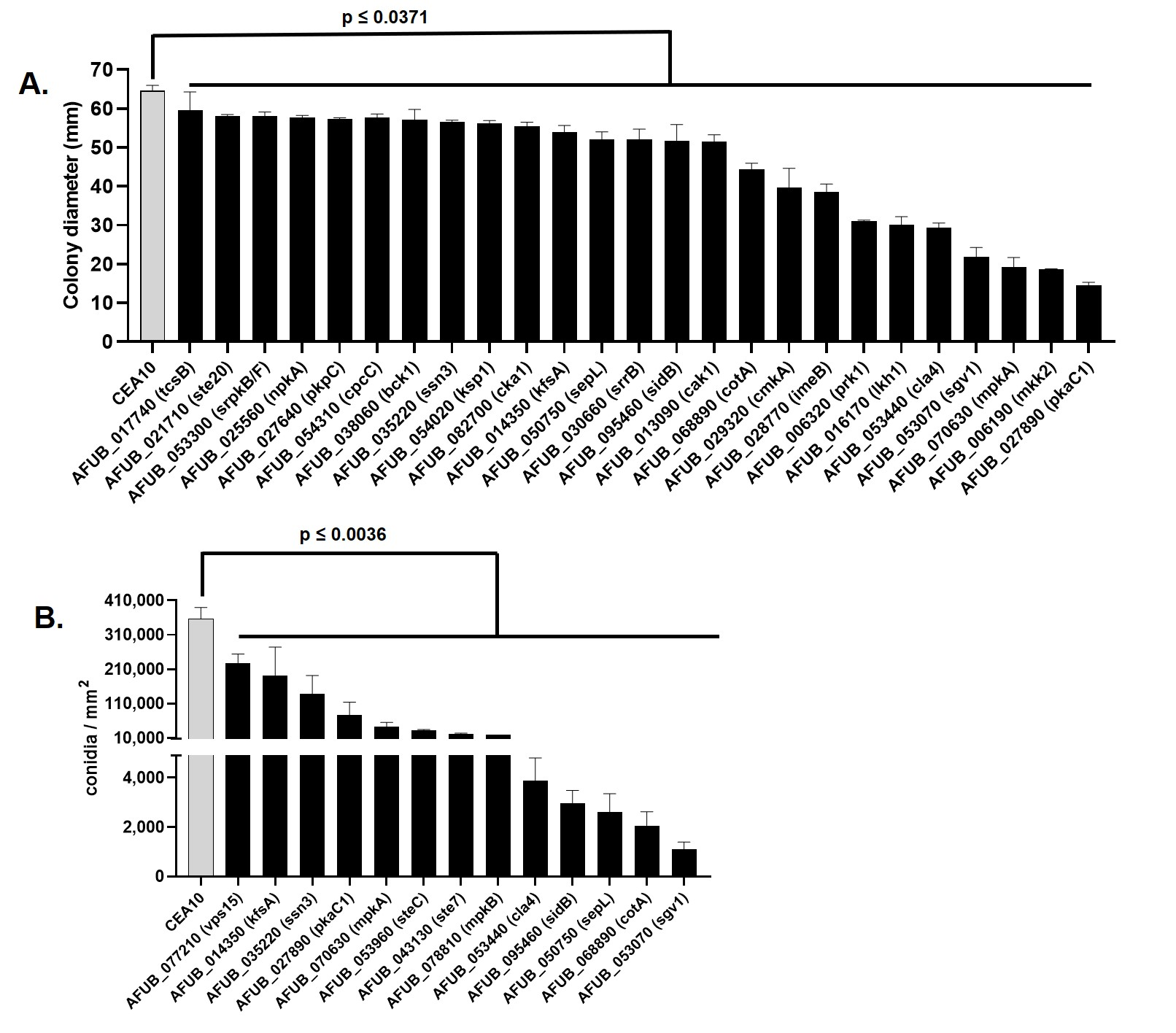

Supplement: S1 Fig — A) Quantitation of colony diameters for the wild type parent strain (CEA10) and disruption mutants displaying minimal, moderate or severe growth restriction on minimal media. 10,000 conidia from each strain were point-inoculated onto minimal media and cultured for 96 hrs at 37°C. Colony diameters form triplicate cultures for each strain were measured (mm) and averaged. Statistical comparisons were made by ANOVA and all comparisons generated a p ≤ 0.0371. Disruption mutants not shown generated colony diameters that were similar to CEA10. B) Quantitation of conidiation for the parent strain (CEA10) and multiple protein kinase gene disruption mutants. Conidia (2 x 10^4^) from each strain were cultured as in (A). Colony area was calculated and conidia were harvested in 10 ml of sterile water before filtration and quantitation using a hemocytometer. Each strain was assayed in triplicate and data were averaged. Statistical comparisons were made by one-way ANOVA with Dunnett’s multiple comparisons post hoc and all comparisons generated a p ≤ 0.0036. Disruption mutants not included here for colony diameter or conidiation analyses were not significantly different from the parental strain (CEA10). (TIF) [file ppat.1009806.s001.tif]

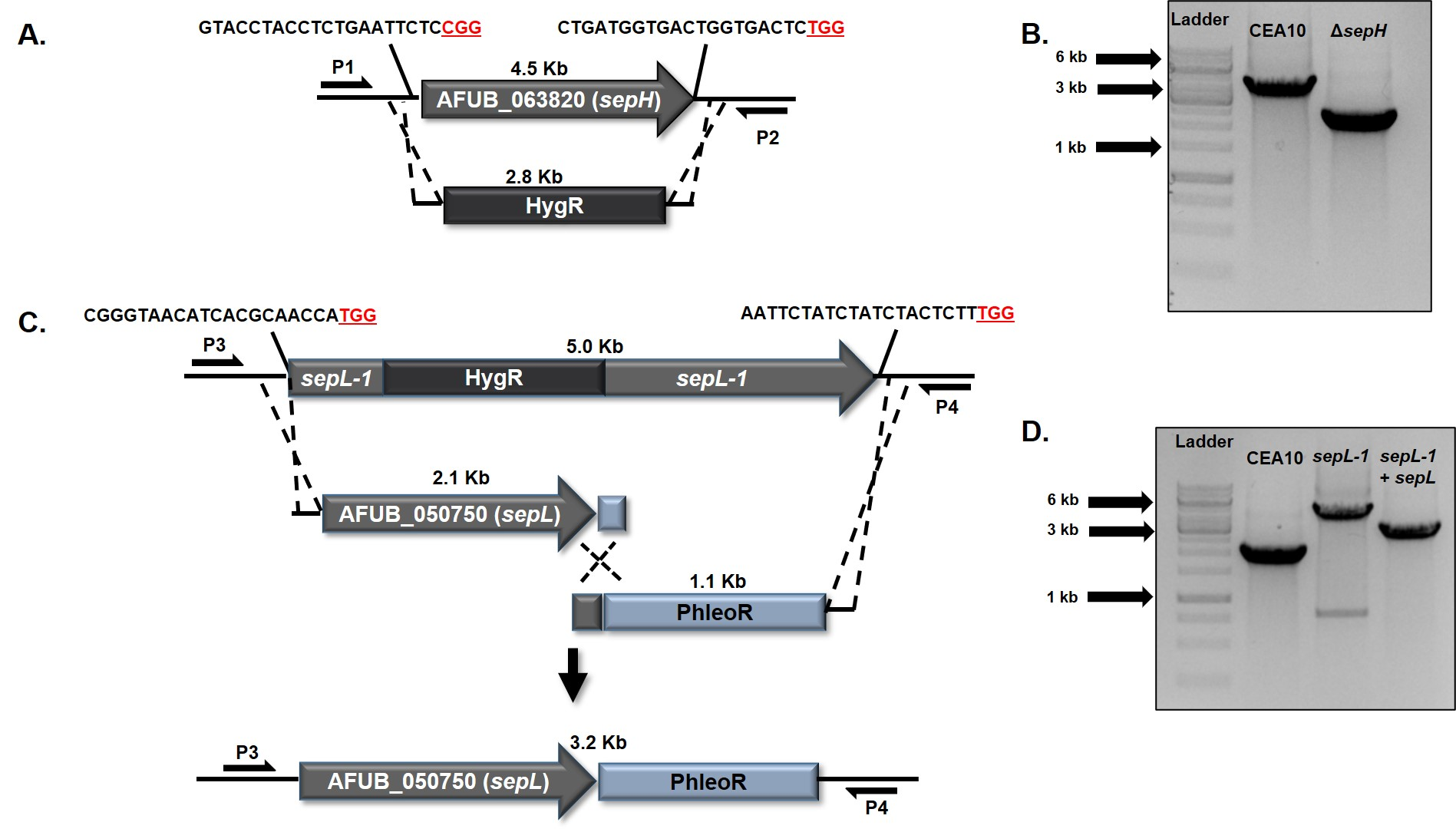

Supplement: S2 Fig — Schematics for deletion of sepH (A) and for complementation of the sepL-1 disruption mutant (C). Genetic manipulations were carried out using CRISPR/Cas9 gene editing (see Materials and Methods). For each locus targeted, the 20-nucleotide protospacer (black font) and the 3-nucleotide protospacer adjacent motif (PAM, underlined red font) are displayed. Each manipulation utilized Cas9-mediated double strand breaks generated 5’ and 3’ of the targeted gene. Repair templates (HygR = hygromycin resistance cassette; PhleoR = phleomycin resistance cassette) were PCR amplified from plasmids using primers that incorporated 40-basepair microhomology arms for targeting. Correct integration of repair templates was confirmed by PCR using primers P1 and P2 for sepH deletion (B) and primers P3 and P4 for sepL-1 complementation (D). (TIF) [file ppat.1009806.s002.tif]

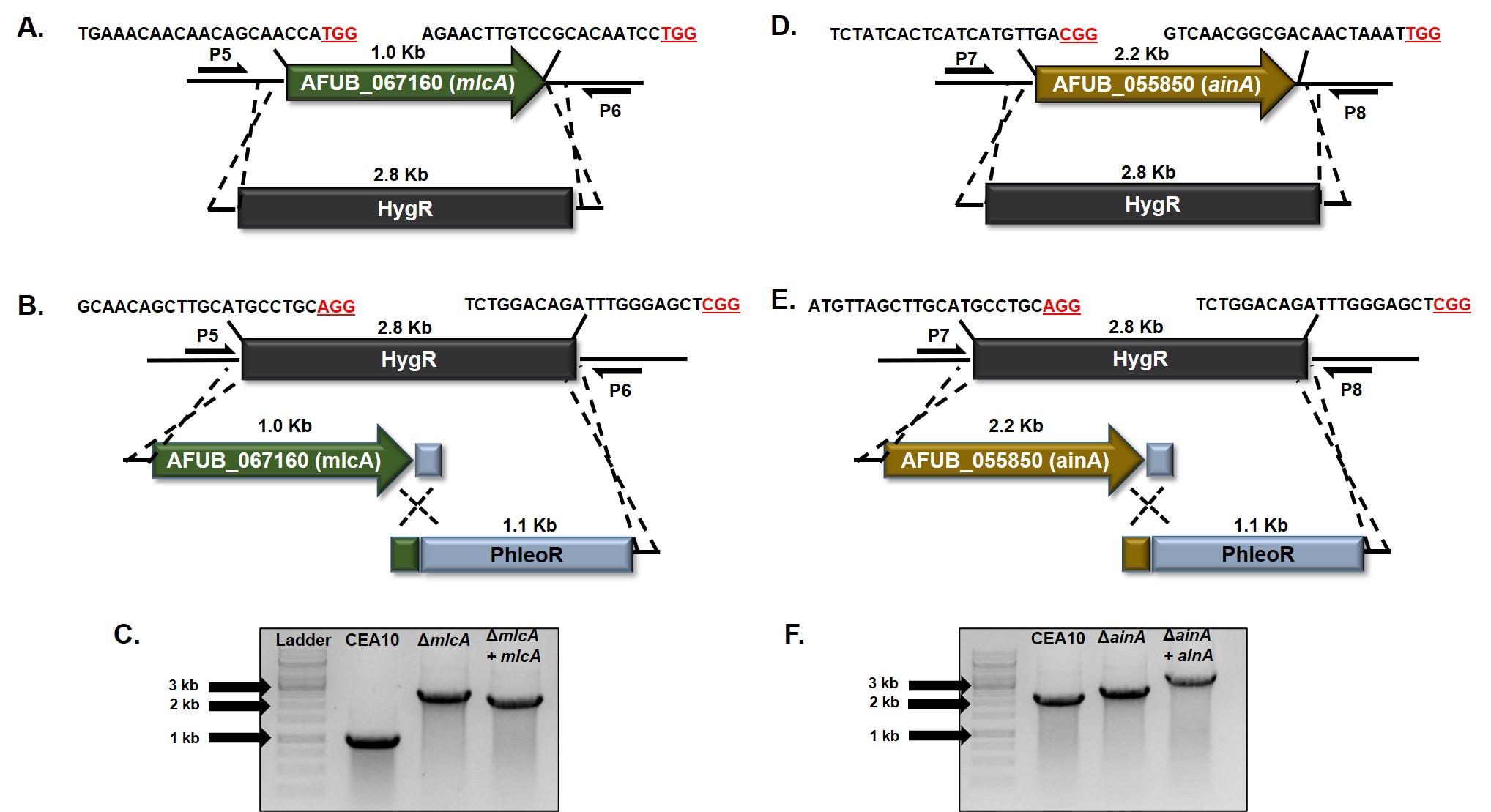

Supplement: S3 Fig — Schematics for deletion (A and D) and complementation (B and E) of mlcA and ainA, respectively. Genetic manipulations were carried out using CRISPR/Cas9 gene editing (see Materials and Methods). For each locus targeted, the 20-nucleotide protospacer (black font) and the 3-nucleotide protospacer adjacent motif (PAM, underlined red font) are displayed. Each manipulation utilized Cas9-mediated double strand breaks generated 5’ and 3’ of the targeted gene. Repair templates (HygR = hygromycin resistance cassette) were PCR-amplified from plasmids using primers that incorporated 40-basepair microhomology arms for targeting. Correct integration of repair templates and gene complementations were confirmed by PCR using primers P5 and P6 for mlcA (A and B) and primers P7 and P8 for ainA (E and F). (TIF) [file ppat.1009806.s003.tif]

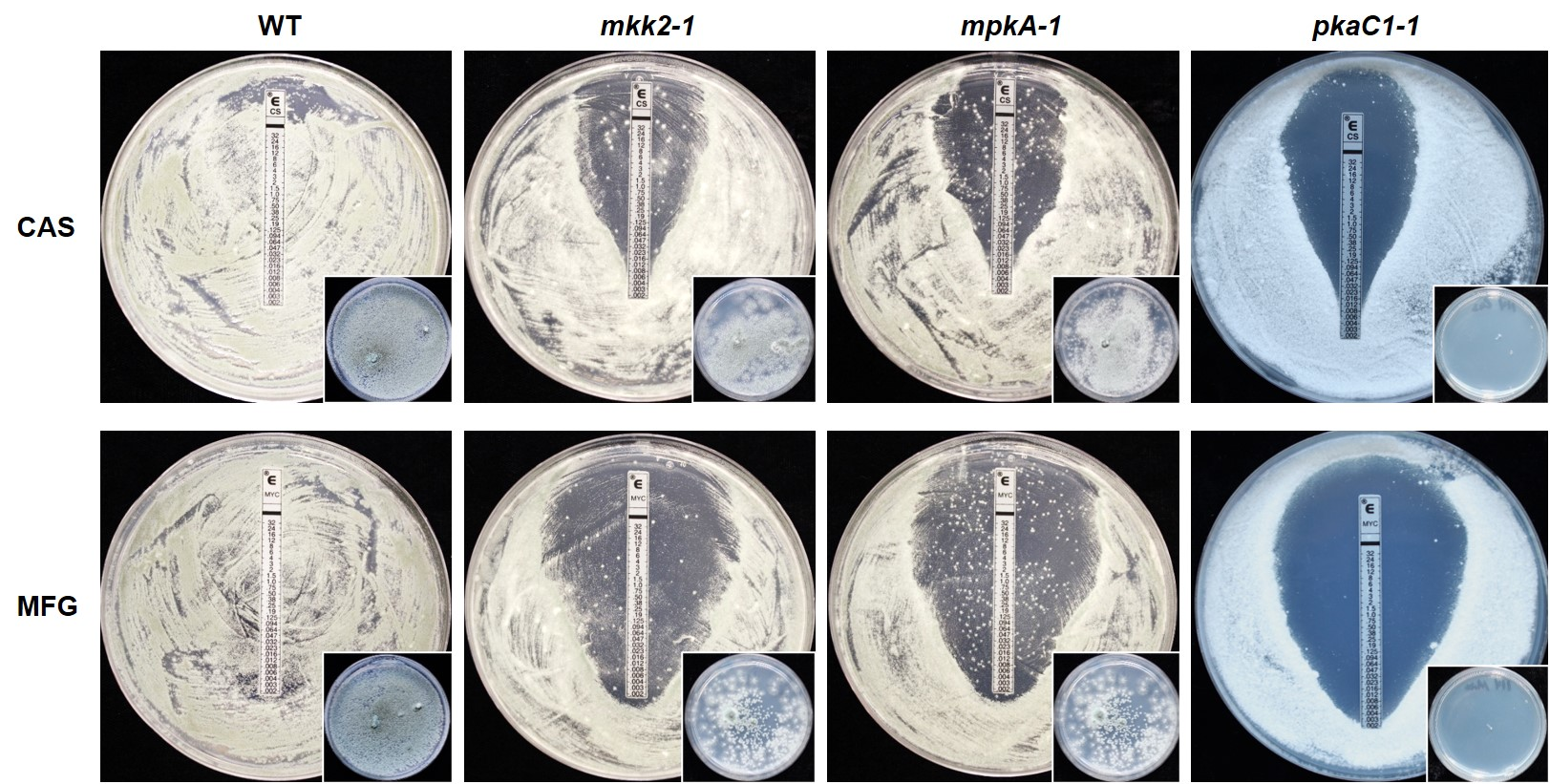

Supplement: S4 Fig — Modified E-test assays for the mkk2-1, mpkA-1, and pkaC1-1 disruption mutants using minimal media (see Materials and Methods). Note the residual growth in the zone-of-clearance for both mkk2-1 and mpkA-1 mutants indicating lack of echinocandin cidal activity. Insets show representative, drug-free minimal media culture plates onto which a single agar plug from the zone-of-clearance for each assay was sub-cultured. Multiple agar plugs (n = 10), taken from within 1 cm of the E-test strip and between the 32 and 0.25 μg/ml markers, were sub-cultured in the same manner for each assay. CAS = caspofungin; MFG = micafungin. (TIF) [file ppat.1009806.s004.tif]
