# Supplementary material for: Loss of Septation Initiation Network (SIN) kinases blocks tissue invasion and unlocks echinocandin cidal activity against Aspergillus fumigatus
Source: PLoS Pathog. 2021 Aug 9;17(8):e1009806. doi: 10.1371/journal.ppat.1009806 (PMC8376064; doi:10.1371/journal.ppat.1009806)
Supplement: Attachment — Submitted filename: Response to Reviewers.docx [file ppat.1009806.s006.docx]

**Response to Reviewers**

All Line numbers reference positions of revisions in the “Track Changes” document.

**Part I - Summary**

**Reviewer #3:**

**Comment:** My only real comment is understanding the link between virulence/resistance and septation per se vs septation-associated biological processes. While I would like to believe that septation is what saves Aspergillus fumigatus from candins, and the data presented is certainly is compatible with this, we know that C. albicans can form septae, yet is highly susceptible to the cidal activity of these agents - so it seems that septae are not a guaranteed way to resist these agents. The obvious question that remains unanswered is - what are the effects of these deletions on the composition of the cell wall other than affecting septation? It looks to me like the sepH mutant has increased CFW staining, and the altered sensitivity to other cell wall agents suggests there are fundamental changes to the cell wall for these mutants.

**Response:** This is an excellent question and we thank the reviewer for their thoughts. We too have speculated as to the potential septation-independent roles of the SIN kinase mutants as related to the phenotypes we have described. As also noted in response to Reviewer #3’s comment in the “Major Issues” section below, we feel that exploration of connections between the septation machinery and the cell wall integrity / biosynthesis machinery deserve a separate, in-depth study to truly delineate mechanism. As such, these studies are outside the scope of the current study. We have included additional wording in the manuscript to clearly state that our data cannot rule out these cell wall-dependent and septation-independent mechanisms. Lines 615-626.

**Part II – Major Issues: Key Experiments Required for Acceptance**

**Reviewer #1:**

**Comment:** I realize that the focus is on echinocandins, but I think the echinocandin results really beg the question about other antifungal classes. Is there any hypersensitivity to nikkomycin or triazoles?

**Response:** We thank the reviewer for this question and we do already have data regarding triazole antifungals. We have noted no changes in susceptibility among the SIN kinase mutants to triazole antifungals by CLSI broth microdilution assays. This information has been added to the text. Lines 325-327. We have not yet utilized nikkomycin Z in our experiments but will do so moving forward as we look to define links between septation and cell wall biosynthesis.

**Reviewer #2:**

**Comment:** Given the plethora of novel and relevant data, relevance of this manuscript is clearly seen by this reviewer. Two interlinked scientific parts are presented, the rather shallow characterization of more than 100 protein kinase mutants that had been generated by an innovative semi-high-throughput approach, to be followed by extensive phenotyping of putative SIN kinase disruptants and regulators of hyphal septation. To fully exploit the range of insights that might be gained from the presented mutants and their phenotypes, some further lines of research could be followed and experimental tasks executed, such as biochemical studies on the presumed kinase targets and their phosphorylation or exploring the postulated positive effect of suited kinase inhibitors or septum formation inhibitors on echinocandin activity (static vs. cidal) and therapy. Yet, the most interesting question arises from the striking phenotype of avirulence that is displayed by the SIN kinase mutants upon pulmonary infection of susceptible mice: Presuming that this results from hampered tissue invasion, one wonders about the outcome in a systemic infection setting…

**Response:** We are in complete agreement with the reviewer regarding the essential next steps for not only the SIN kinase-regulated pathway(s) focused on in our manuscript, but for each protein kinase represented in our mutant library. Biochemical studies to identify kinase target proteins and the regulatory outcome of differential phosphorylation will be a focus for our lab for many years to come. We presume that the reviewer is wondering about how these mutants would perform in a tail vein injection model vs. our pulmonary model. We agree that, although both mouse models used in our study permit dissemination of *Aspergillus* from the lung (especially the chemotherapeutic model), our data does not necessarily provide this piece of information as the SIN kinase mutants appear mostly confined to the pulmonary space. Although there could certainly be value in tail vein injection studies, these studies rely on the unnatural injection of conidia into the bloodstream whereas the route of infection that leads to systemic disease in the human host is understood to be pulmonary. We plan to pursue the reviewer’s suggestion in future studies using doxycycline repressible SIN kinase strains that allow us to initiate invasion and then induce loss of septation with doxycycline (mimicking septation inhibitors as therapy). These studies will still permit us to utilize a natural infection route and also to analyze the impacts of loss of septation on systemic disease and dissemination to distal sites after lung invasion has begun.

**Reviewer #3:**

**Comment:** I would suggest doing an analysis of the cell wall glycan composition of these mutants (with and without echinocandins), at least to confirm if the standard cell wall integrity responses are compromised, and if there are fundamental changes in glycans that could contribute to the observations in the paper. If this is not done, I would suggest a careful rewrite of the discussion to emphasize that while clearly the SIN kinases are required for all the observed phenotypes (and dramatically so!), the current experiments do not distinguish between septation per se, and other SIN kinase-dependent process, as potential mechanisms underlying the findings.

**Response:** We thank the reviewer for this comment. We have detailed plans to analyze the cell wall of each SIN kinase mutant for future studies wherein we will delineate connections between septation and cell wall biosynthesis and in turn decipher mechanistically how this impact tissue invasion and initiation of host responses. In the Discussion of the original submission (Lines 561-574), we did briefly discuss alternative hypotheses as to why the septation mutants may produce specifically some of the noted cell wall phenotypes. As stated, we do believe that there may be some septum-independent mechanisms at play here especially given the varying abilities of the Sin kinase mutants to induce pro-inflammatory host responses. To ensure that we clearly state that our data cannot rule these possibilities out, we have more directly stated that our current experiments do not distinguish between septation and other processes (as suggested by the reviewer). Lines 615-626.

**Part III – Minor Issues: Editorial and Data Presentation Modifications**

**Reviewer #1:**

**Comment:** It is interesting that similar mutants in a plant pathogen reduce turgor pressure necessary for appressoria, raising the possibility that the lack of invasiveness is a related problem. Will the mutants grow on explants of lung tissue tissue on plates similar to how they behave on minimal medium? If they can’t invade the substrate, perhaps not. This could get complicated, so perhaps experiments along these lines would be best left to a follow-up study that delves into the invasive properties of the hyphae.

**Response:** We agree and think this is an excellent suggestion for deciphering our lack-of-invasion phenotype moving forward. A positive or negative growth phenotype resulting from this experiment could be interpreted in many ways and would certainly require more mechanistic follow up. The lack of invasion may require a living host and/or invasive growth initiation to occur on the lung epithelial surface making data interpretation difficult as a standalone experiment. In addition, more experimental evidence is being sought to connect (or separate) septation pathways and pathways regulating cell wall biosynthesis or secretion. Pathways controlling both of these would impact the ability of the SIN kinase mutants to grow on explants. Therefore, we plan to perform these experiments in follow up studies.

**Comment:** Are the mutants temperature sensitive? This is not a major issue since the focus of the ms is on pathogenesis and hence 37°C is the most relevant. However, I would be curious to see it mentioned in the discussion if the results are already available.

**Response:** We have not yet performed a comprehensive analysis of temperature sensitivity but agree this is an interesting and potentially very informative phenotype to characterize. As stated above, we plan to do these experiments moving forward as we characterize the links between the septation machinery, cell wall construction and secretion.

**Comment:** Line 67 – is limited by patient toxicity and threatened by resistance

**Response:** Corrected.

**Reviewer #2:**
**Comment:** Significant parts of the Introduction are spent on describing results (e.g., l. 99-106 or l. 121-131). Maybe providing more context with respect to invasive(!) aspergillosis and knowledge about virulence determinants thereof might be illustrative without giving away too many findings.

**Response:** We have made revised and reorganized the Introduction to reduce all presentation of current results to the final paragraph and have extended the first section to introduce the process of invasive aspergillosis. Lines 74-85. As many genes have been described as generating mutants with reduced virulence in IA models, and many of these do not utilize or invade tissue well, as summary of virulence determinant in the Introduction would, we feel, be long and distractive.

**Comment:** In the Results section, it is puzzling why the disruption library was established in the CEA10 wild-type isolate after identifying kinase-encoding orthologues in the genome sequences of A1163 and Af293 (l. 137-145) – please clarify for the readers that are not familiar with A. fumigatus laboratory isolate lineages.

**Response:** We apologize for the confusion and further clarified the fact that the library was established in the same genetic background in which the kinase genes were identified. Comparisons to Af293 were performed to ensure we were not studying strain-specific differences in putative kinase genes. Lines 165-173.

**Comment:** What might be the reason for the unexpected observation that several protein kinase mutants display a mild growth reduction that has not, with the exception of bck1, been described for any of the corresponding A. fumigatus mutants before (l. 185-193)?

**Response:** We thank reviewer for picking up on this. The other mutants that have been previously studied that fit in to this growth reduction category were all deletion mutants (not disruption mutants as we have generated here that may represent partial loss-, total-loss- or even gain-of-function) and were generated in different strain backgrounds. In addition, for most of these deletion mutants, basal growth attributes were not reported and rich media was used for testing. Therefore, many differences in experimental approach likely resulted in the finding that only bck1 mutation was previously reported to induce a growth defect. To eliminate any confusion and to avoid making poor comparisons, we have removed this statement.

**Comment:** What is the actual mode of action for the cell wall stressors Calcofluor White and Congo Red (l. 208/9)?

**Response:** We have included additional text (with reference) to define the mode of action for both agents. Lines 239-242.

**Comment:** The notion that some of the library strains might not be altered in the respective gene function is puzzling (l. 219/202) – to what extent was this checked for the bck1 gene that gave rise to this suspicion albeit displaying a growth phenotype?

**Response:** We agree that it would be excellent if all gene disruptions produced only loss-of-function phenotypes. However, our disruption scheme did not necessarily attempt destroy the kinase domain (or other functional domains) of each resulting protein. This would only occur through loss of gene transcription or disruptive frameshift due to insertion of our disruption repair template. Therefore, any resulting mutant strain that did produce one of these genetic outcomes due to aberrant repair template integration could still potentially produce a kinase that was (at least) partially functional. We have not yet examined the entire library for the frequency of such an occurrence and we did not follow this up to any more detail for bck1 than what is described.

**Comment:** Hyphal damage of SIN kinase mutants in the presence of micafungin is demonstrated by PI permeability assays (l. 319-330), which could be further supported by leakage experiments using strains that express fluorescent proteins.

**Response:** We agree that additional work would further support the hyphal damage phenotype but feel that, when taken in total, our data clearly show that loss of septation leads to the inability of hyphae to resist cell wall damage by limiting the extent of damage. As described in the original submission, this conclusion is well-supported by the well characterized role of septa in protection against hyphal damage. We have planned future work that will utilize tagged strains to analyze the kinetics of hyphal damage in not only the SIN kinase mutant strains, but other cell wall-regulating kinases.

**Comment:** Description of the virulence phenotypes of SIN kinase mutants (l. 349-352) is somehow unclear.

**Response:** We apologize for this sentence being worded in an unclear manner and have corrected it.

**Comment:** When confirming dependency of IL-1beta secretion on NLRP3 inflammasome activity (l. 380-385), citing one of the most recent literature on this rather distractive issue (e. g., PMID: 33268895) might be convenient.

**Response:** We have now cited the reference suggested by the reviewer, as well as the original reference describing NLRP3 inflammasome activation by *Aspergillus*. Lines 412-414.

**Comment:** The Discussion’s reasoning that viable fungal elements are especially problematic for neutropenic patients (l. 556-557) is somehow awkward.

**Response:** We apologize for the awkward wording of this sentence. The term “fungal elements” is clinical phrasing for the identification of presumed fungal hyphae and/or conidia in patient samples. We have corrected the sentence for greater clarity.

**Comment:** When speculating on the lack of turgor pressure in the tip compartment being the underlying mechanisms for the observed virulence and tissue invasion phenotypes, recent insights deduced from microfluidic experimentations (PMID: 33727355) could be incorporated.

**Response:** We thank the reviewer for bringing our attention to this very interesting new publication. We agree that it raises excellent points for speculation about the roles that septation may play in imposing the tradeoffs between hyphal growth rate, turgor pressure and maintenance of polarity during tissue invasion. We have now included a brief statement regarding this and referenced this manuscript. Lines 606-615.
